# Dr. J. V. C. Smith

**Published:** 1851-10

**Authors:** 


*Dr. J. V. C. Smith.*—We have been somewhat tardy in noticing the return of our distinguished *confrere*, the editor of the Boston Medical and Surgical Journal, but our congratulations are not less cordial than if they had been more promptly rendered. During an absence of several months, Dr. Smith has visited Europe, Asia, Africa, and, in the mean time, the readers of his valuable Journal have been favored with highly interesting letters written at various points in his extensive tour. Of a portion of this correspondence we have availed ourselves for the benefit of our readers, and we should have done so more largely had the original matter contributed for our pages been less in amount. We presume the letters will be published separately, and they will make an interesting volume.

In resuming his editorial duties, Dr. Smith remarks, that "Diseases are no where controlled with more skill than in the United States, nor is there a country on the globe where the benefits accruing to society from the efforts of indefatigable practitioners of medicine and surgery are more apparent." It must be pleasant for the medical traveler to return with such convictions, and they are certainly gratifying to those who are obliged to remain at home, and relinquish to others the agreeable task of studying the men and manners of other countries.

